# Serum marker and CT characteristics of coronary calcified nodule assessed by intravascular ultrasound

**DOI:** 10.1186/s12872-022-02931-z

**Published:** 2022-11-09

**Authors:** Jing Li, Jing Li, Zhijie Jian, Jianhua Wu, Jian Yang, Ning Guo, Xin Huang

**Affiliations:** 1 Department of Cardiology, the First Affiliated Hospital of Xi’an Jiaotong University, Xi’an, Shaanxi, 710061 P.R. China; 2 Department of Radiology, the First Affiliated Hospital of Xi’an Jiaotong University, Xi’an, Shaanxi, 710061 P.R. China

**Keywords:** Calcified nodule, Coronary calcification, Intravascular ultrasound, Computed tomography angiography, Alkaline phosphatase

## Abstract

**Background:**

Calcified nodule (CN) is a type of potentially vulnerable plaque. Its formation mechanism remains unknown. This study was to assess serum marker and computed tomography angiography (CTA) characteristics of CN validated by intravascular ultrasound (IVUS).

**Methods:**

Patients who underwent coronary CTA followed by invasive coronary angiography and IVUS were retrospectively analyzed. Serum levels of alkaline phosphatase (ALP), gamma-glutamyltransferase, and calcium were collected.

**Results:**

IVUS detected 128 de novo calcified lesions in 79 patients with coronary artery disease (CAD). CNs were identified in 11.4% (9/79) of patients and 9.4% (12/128) of lesions. Compared with patients with non-nodular calcified plaques, CN patients had higher serum level of ALP (82.00 vs 65.00 U/L, *P* = 0.022) and total plaque volume (673.00 vs 467.50 mm^3^, *P* = 0.021). Multivariable analyses revealed that serum ALP level and total plaque volume were independently associated with the prevalence of CN in CAD patients with calcified plaques. At lesion level, the CN group had a higher frequency of moderate to heavy calcification on angiography (75.00% vs 40.52%, *P* = 0.017). In terms of CTA characteristics, plaques with CN had a more severe diameter stenosis (79.00% vs 63.00%, *P* = 0.007), higher plaque burden (85.40% vs 77.05%, *P* = 0.005), total plaque density (398.00 vs 283.50 HU, *P* = 0.008), but lower lipid percentage (14.65% vs 19.75%, *P* = 0.010) and fiber percentage (17.90% vs 25.65%, *P* = 0.011). Mean plaque burden is an independent predictor of the prevalence of CN in calcified plaques (odds ratio = 1.102, 95% confidence interval: 1.025–1.185, *P* = 0.009). The AUC is 0.753 (95% confidence interval: 0.615–0.890, *P* = 0.004). When using 84.85% as the best cutoff value, the diagnostic sensitivity and specificity of mean plaque burden for predicting the presence of CN within calcified plaques were 66.7% and 80.2%, respectively.

**Conclusions:**

CN had different CTA imaging features from non-nodular coronary calcification. The presence of a CN was associated with a higher serum ALP level and plaque burden.

## Background

Non-invasive imaging offers a screening tool to identify plaques with features of vulnerability, stratifying patients at increased cardiovascular risk. The coronary calcified nodule (CN) is suggested as a potentially vulnerable plaque accounting for approximately 2% to 7% of coronary events with worse clinical outcomes [1]. CN on intravascular ultrasound (IVUS) is an eruptive, dense, calcified mass that usually has an irregular surface appearance [2]. However, there is limited information about its serum marker and features on computed tomography angiography (CTA) imaging. The aim of this study was to assess the serum marker and CTA characteristics of CN validated by IVUS.

## Methods

### Study population

**Fig. 1:**
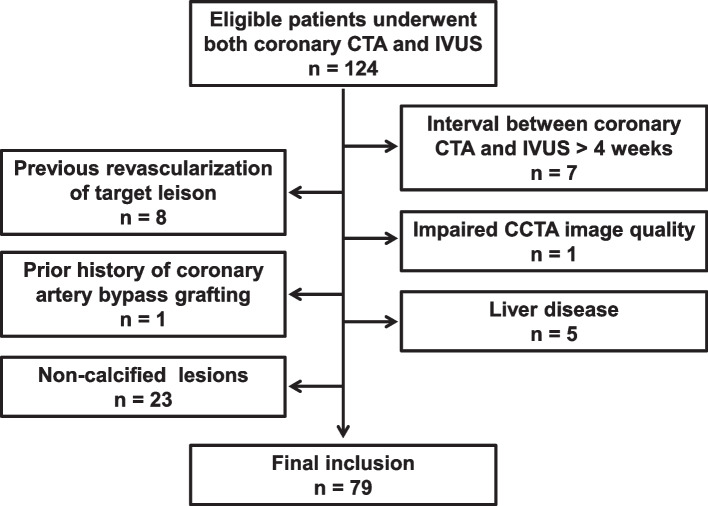
Flow chart of patient inclusion and exclusion criteria. CTA, computed tomography angiography; IVUS, intravascular ultrasound

Consecutive patients with stable angina or angina-equivalent symptoms of intermediate CAD pretest probability referred for coronary CTA to exclude obstructive coronary stenosis at the First Affiliated Hospital of Xi'an Jiaotong University from Jan 2018 to Dec 2019 were retrospectively reviewed. Patients who underwent both coronary CTA and IVUS were screened and included. The main indication for IVUS was to evaluate lesions and guide percutaneous coronary intervention (PCI). The inclusion criteria were CTA and IVUS were performed to assess native coronary lesions within 4 weeks. Exclusion criteria were: (1) impaired CTA image quality; (2) previous revascularization of target lesion; (3) prior history of coronary artery bypass grafting; (4) patients with liver disease; (5) patients of coronary non-calcified lesions (Fig. 1).

Data regarding demographic properties and laboratory parameters, including serum levels of alkaline phosphatase (ALP), gamma- glutamyltransferase, and calcium of the patients, was collected from medical records. Fasting blood samples were collected within 24 h after admission, and serum levels of ALP, gamma-glutamyltransferase, and calcium were analyzed routinely by an automated commercial kit (Boehringer Mannheim, Mannheim, Germany). Chronic kidney disease was defined as glomerular filtration rate of < 60 mL/min per 1.73 m^2^.

### Coronary CTA acquisition

All patients underwent ECG-gated CTA using a 256-slice computed tomography scanner (GE Healthcare, Shanghai, China). A standard scanning protocol was performed with section collimation of 2 × 128 × 0.625 mm, tube current between 200 and 360 mA at 120 kV, and gantry rotation time of 270 ms. A weight-dependent bolus of 70–90 ml iodine contrast agent (iohexol, 350 mg iodine/ml) was injected into the antecubital vein at a rate of 4 to 5.5 ml/s using a dual-head injector. Cardiac CT images were reconstructed at 75% and 45% of the RR interval.

### Assessment of coronary plaques

CTA is a reliable tool for plaque characterization and quantification including calcium [3]. All coronary CTA data was analyzed using a Vitrea workstation (Vital Images, Minnetonka, Minnesota). Plaques were defined as structures > 1 mm^2^ within and/or adjacent to the vessel lumen. Minimal lumen diameter, reference vessel diameter, lesion length, and plaque volume were measured. Mean plaque burden was defined as the aggregate plaque volume divided by the total vessel volume. A remodeling index was defined as a maximal lesion vessel diameter divided by proximal reference vessel diameter, with positive remodeling defined as a remodeling index ≥ 1.1. Semi-automated compositional analysis was performed using Hounsfield units (HU) cut-off values of -100 to 49 for lipid, 50 to 149 for fiber, and 150 to 1300 for calcium. Each component volume (mm^3^) and the percentage is calculated automatically based on all voxels within the selected coronary segment. Two experienced cardiovascular radiologists who were blinded to IVUS data and each other's assessment (JY and ZJJ) measured the characteristics of CTA separately. The average of both readings was used for the final analysis.

### Intravascular ultrasound

**Fig. 2:**
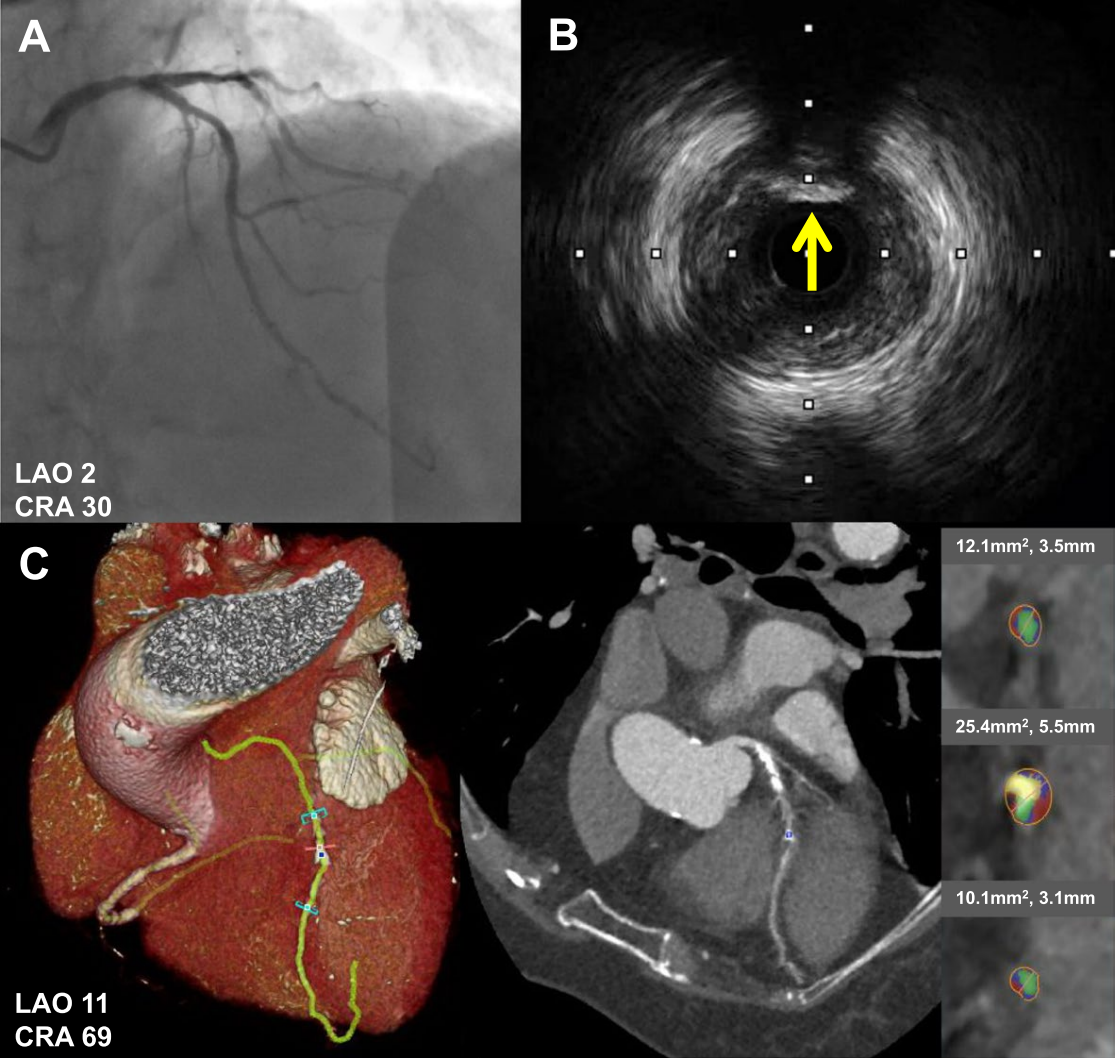
Representative case of quantitative coronary computed tomography angiography (CTA) analysis in patient with calcified nodule. **A** Invasive angiography showed intermediate coronary stenosis at the proximal to middle left anterior descending. **B** Intravascular ultrasound confirmed the presence of calcified nodule (yellow arrow). **C** CTA imaging of plaque with calcified nodule. On the color-coded cross-sectional image, lipid was labeled as red, fiber was labeled as blue and calcium was labeled as yellow

Based on the angiographic findings, if clinically requested, IVUS examinations were performed prior to percutaneous coronary intervention in a standard fashion with the catheter (40 MHz Opticross catheter, Boston Scientific, USA) during automatic pullback at a speed of 0.5 mm/s. The target lesions of IVUS imaging are ischemia-related lesions judged by angiographic results and clinical data. Off-line IVUS analyses were performed using QIvus 2.1 software (Medis medical imaging systems, the Netherlands) in consensus by two observers (NG and XH) blinded to all other information of the same patient. CN was defined as calcification with an irregular, protruding, and convex luminal surface (Fig. 2B) [2].

### Statistical analysis

Normally distributed continuous variables were expressed as mean ± SD, non-normal distribution as median with interquartile range (IQR), and categorical variables as numbers and percentages. For comparison between groups, independent samples t-test or Mann–Whitney U test were applied for continuous variables and the χ2 test or Fisher's exact test for categorical variables as appropriate. Multivariate analyses with a forward stepwise approach were performed to identify the factors that were independently associated with the prevalence of CN. The receiver operating characteristic (ROC) curve analysis was applied to evaluate the predictive performance of the mean plaque burden for CN. The optimal cut-off point was determined as the value of the maximum Youden index. A statistically significant difference was defined as a 2-sided *P* value < 0.05. Statistical analyses were performed using SPSS (version 26.0, SPSS Inc., Chicago, IL, USA).

## Results

### Baseline clinical characteristics

A total of 124 patients who underwent both coronary CTA and IVUS were initially reviewed. Seven patients with the interval between CTA and IVUS longer than 4 weeks, 1 patient with impaired CTA image quality, 8 patients with the previous revascularization of the target lesion, 1 patient with prior history of coronary artery bypass grafting, 5 patients with liver disease were excluded. Twenty-three patients were also excluded due to non-calcified coronary lesions (Fig. 1). Finally, 79 patients with 128 lesions were included in the present study.

Figure 2 shows a representative case of CTA plaque component analysis in a patient with CN. CNs were identified in 11.4% (9/79) of patients and 9.4% (12/128) of lesions. Baseline clinical characteristics comparing 9 patients with CN and 70 patients with non-nodular calcification are listed in Table 1. The frequency of diabetes, hypertension, smoking, chronic kidney disease, and acute coronary syndrome (ACS) presentation was statistically similar between the two groups.

**Table 1:** Comparison of baseline clinical and computed tomography angiography characteristics at patient level

	Whole Cohort (*n* = 79)	Calcified nodule (*n* = 9)	Non-nodular calcification (*n* = 70)	*P* value
Age (year)	61.76 ± 11.53	62.11 ± 13.39	61.71 ± 11.38	0.923
≥ 70 year (%)	21 (26.58)	3 (33.33)	18 (25.71)	0.693
Men (%)	62 (78.48)	8 (88.89)	54 (77.14)	0.675
Body mass index (kg/m^2)^	24.94 (23.66, 26.47)	24.94 (23.34, 26.44)	24.94 (23.66, 26.53)	0.609
Current smoking (%)	17 (21.52)	1 (11.11)	16 (22.86)	0.675
Diabetes (%)	28 (35.44)	3 (33.33)	25 (35.71)	1.00
Hypertension (%)	58 (73.42)	7 (77.78)	51 (72.86)	1.00
Dyslipidemia (%)	79 (100.00)	9 (100.00)	70 (100.00)	NC
Prior myocardial infarction (%)	17 (21.52)	2 (22.22)	15 (21.43)	1.00
Prior PCI (%)	16 (20.25)	2 (22.22)	14 (20.00)	1.00
Chronic kidney disease (%)	6 (7.59)	0 (0)	6 (8.57)	1.00
Family history of premature CAD (%)	6 (7.59)	0 (0)	6 (8.57)	1.00
Clinical presentation				
Stable angina pectoris (%)	15 (18.99)	4 (44.44)	11 (15.71)	0.061
Acute coronary syndrome (%)	64 (81.01)	5 (55.56)	59 (84.29)	0.061
Total cholesterol (mmol/L)	3.47 (2.94, 4.26)	3.17 (2.65, 3.79)	3.58 (2.94, 4.32)	0.257
HDL-C (mmol/L)	0.90 (0.77, 1.01)	0.81 (0.76,0.92)	0.91 (0.79, 1.01)	0.396
LDL-C (mmol/L)	2.03 (1.51, 2.57)	1.83 (1.34, 2.28)	2.04 (1.50, 2.70)	0.280
Triglyceride (mmol/L)	1.35 (0.97, 1.81)	1.12 (0.68, 1.57)	1.43 (1.01, 2.01)	0.063
Hemoglobin A1C (%)	6.00 (5.60, 6.90)	6.00 (5.40, 6.58)	6.10 (5.60, 7.00)	0.482
Fasting blood glucose (mmol/L)	5.60 (4.91, 7.06)	5.08 (4.19, 6.28)	5.60 (5.06, 7.18)	0.263
GFR (ml/min/1.73m^2^)	106.21 (87.48, 124.13)	111.06 (87.41, 123.16)	106.13 (88.40, 124.42)	0.853
Peak hs-cTnT (ng/mL)	0.011 (0.006, 0.057)	0.008 (0.0045, 0.0805)	0.011 (0.006, 0.0595)	0.671
Peak pro-BNP (pg/mL)	124.30 (50.22, 637.90)	100.80 (44.48, 240.00)	126.65 (51.93, 728.75)	0.388
hs-CRP (mg/L)	2.85 (1.15, 2.85)	1.31 (0.62, 6.43)	2.85 (1.17, 2.85)	0.634
Alkaline phosphatase (U/L)	69.00 (52.00, 87.00)	82.00 (54.50, 93.50)	65.00 (42.00, 77.50)	0.022
Gamma-glutamyltransferas (U/L)	26.00 (17.00,37.00)	25.00 (18.00, 43.50)	26.00 (16.75, 37.00)	0.920
Serum calcium (mmol/L)	2.25 ± 0.15	2.21 ± 0.14	2.26 ± 0.15	0.336
Left ventricular ejection fraction (%)	67.00 (61.00, 71.00)	70.00 (62.50, 75.00)	67.00 (61.00, 71.00)	0.256
Multi-vessel disease (%)	52 (65.82)	6 (66.67)	46 (65.71)	0.325
Total plaque volume (mm^3^)	490.00 (151.00, 969.00)	673.00 (256.50, 1516.05)	467.50 (150.50, 940.75)	0.021
Lipid volume (mm^3^)	89.90 (40.40, 163.40)	119.30 (48.65, 229.90)	80.50 (40.23, 161.15)	0.468
Fiber volume (mm^3^)	122.80 (54.10, 208.60)	205.10 (64.40, 282.60)	122.25 (53.70, 201.43)	0.347
Calcification volume (mm^3^)	236.60 (52.90, 585.80)	468.50 (144.75, 1029.40)	197.30 (49.38, 582.48)	0.165
Lipid percentage (%)	20.64 (15.97, 27.25)	17.12 (12.44, 21.55)	20.71 (16.25, 29.09)	0.073
Fiber percentage (%)	25.22 (20.39, 39.91)	22.42 (15.65, 31.06)	25.64 (20.50, 40.53)	0.235
Calcification percentage (%)	54.91(41.48, 64.20)	60.36 (47.77, 71.20)	54.58 (39.99, 63.68)	0.156

Normally distributed continuous variables were expressed as mean ± SD, non-normal distribution as median with interquartile range (IQR), and categorical variables as numbers and percentages

*Abbreviations:* *CAD* coronary heart disease, *GFR* glomerular filtration rate, *HDL-C* high-density lipoprotein cholesterol, *hs-CRP* high-sensitivity C-reactive protein, *LDL-C* low-density lipoprotein cholesterol, *LVEF* left ventricular ejection fraction, *PCI* percutaneous coronary intervention, *pro-BNP* pro-brain natriuretic peptide

Patients with CN had higher serum level of ALP (82.00 vs 65.00 mmol/L, *P* = 0.022) and total plaque volume (673.00 vs 467.50 mm^3^, *P* = 0.021) than those with non-nodular calcified plaques. After adjusted for age, current smoking, the history of hypertension, diabetes, and chronic kidney disease, serum ALP level (odds ratio [OR] = 1.008, 95% confidence interval [CI]: 1.004 -1.023, *P* = 0.03) and total plaque volume (OR = 1.006, 95% CI: 1.003 -1.009, *P* = 0.04) remained independently associated with the prevalence of CN in patients with coronary calcified plaques.

### Comparison of angiographic and CTA-derived parameters among CN and non-nodular calcified plaques

At lesion level, the CN group had a higher frequency of moderate to heavy calcification on angiography (75.00% vs 40.52%, *P* = 0.017). Lesions with CN presented a more severe diameter stenosis (79.00% vs 63.00%, *P* = 0.007). As compared with the non-nodular calcified plaques, the CN had higher mean plaque burden (85.40% vs 77.05%, *P* = 0.005) and total plaque density (398.00 vs 283.50 HU, *P* = 0.008), but lower lipid percentage (14.65% vs 19.75%, *P* = 0.01) and fiber percentage (17.90% vs 25.65%, *P* = 0.011). The plaques with CN have similar calcium percentage (64.20% vs 54.35%, *P* = 0.055) and calcium density (530.00 vs 495.50 HU, *P* = 0.277) with the non-nodular calcific ones (Table 2).

**Table 2:** Comparison of angiographic and computed tomography angiography characteristics at lesion-level

	Calcified nodule (*n* = 12)	Non-nodular calcification (*n* = 116)	*P* value
Lesion location (%)			
LAD	7 (58.33)	63 (54.31)	0.790
LCX	3 (25.00)	32 (27.59)	1.000
RCA	2 (16.67)	21(18.10)	1.000
Initial TIMI flow grade 0–1 (%)	1 (8.33)	21 (18.10)	0.684
Moderate to heavy calcification on angiography (%)	9 (75.00)	47 (40.52)	0.017
Type B2/C lesion (%)	8 (66.67)	68 (58.62)	0.720
Plaque computed tomography angiography analysis			
Minimal lumen diameter (mm)	0.45 (0.35, 2.70)	0.95 (0.25, 2.74)	0.089
Reference vessel diameter (mm)	3.55 (3.23, 4.02)	3.36 (2.97, 3.89)	0.350
Diameter stenosis (%)	79.00 (52.00, 76.00)	63.00 (39.02, 60.00)	0.007
Lesion length (mm)	23.85 (13.83, 44.93)	19.75 (13.50, 31.70)	0.416
Positive remodeling (%)	0	24 (20.69)	0.121
Mean plaque burden (%)	85.40 (77.85, 91.85)	77.05 (65.63, 82.60)	0.005
Total plaque volume (mm^3^)	378.00 (150.00, 870.00)	290.00 (146.50, 520.75)	0.364
Lipid volume (mm^3^)	66.55 (31.78, 102.78)	57.40 (36.03, 92.25)	0.915
Fiber volume (mm^3^)	90.40 (39.70, 110.68)	86.30 (48.23, 131.28)	0.731
Calcium volume (mm^3^)	227.55 (80.03, 626.60)	151.30 (53.78, 308.83)	0.236
Lipid percentage (%)	14.65 (12.70, 17.85)	19.75 (16.03, 25.40)	0.010
Fiber percentage (%)	17.90 (14.08, 27.13)	25.65 (20.88, 37.55)	0.011
Calcium percentage (%)	64.20 (46.80, 72.35)	54.35 (38.73, 62.50)	0.055
Total plaque density (HU)	398.00 (301.00, 452.75)	283.50 (188.75, 352.75)	0.008
Lipid density (HU)	-3.00 (-9.75, 6.50)	0.00 (-6.00, 5.75)	0.559
Fiber density (HU)	99.50 (98.00, 101.00)	98.00 (96.00, 100.00)	0.066
Calcium density (HU)	530.00 (437.75, 599.75)	495.50 (389.50, 548.25)	0.277

Normally distributed continuous variables were expressed as mean ± SD, non-normal distribution as median with interquartile range (IQR), and categorical variables as numbers and percentages

*Abbreviations:* *HU* Hounsfield Units, *LAD* left anterior descending, *LCX* left circumflex, *RCA* right coronary artery, *TIMI* thrombolysis in myocardial infarction

### Diagnostic performance of CTA-derived mean plaque burden for predicting the presence of a CN

**Fig. 3:**
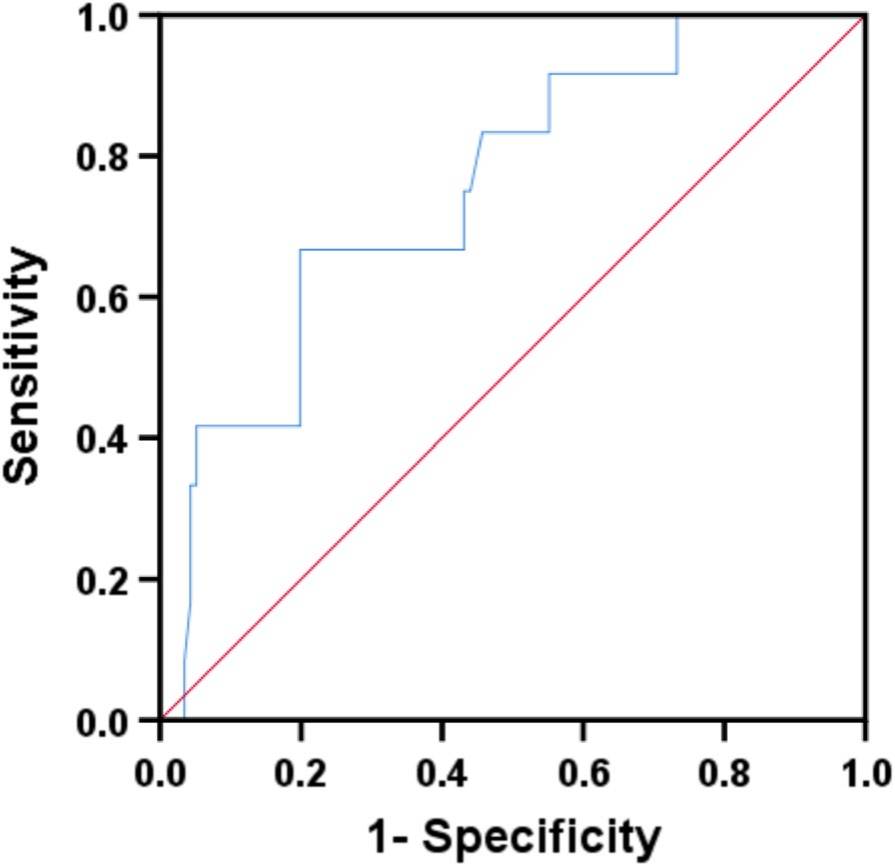
Receiver operating characteristic analysis of plaque burden per vessel for predicting calcified nodules in calcific plaques (area under the curve 0.753, 95% CI: 0.615–0.890, *P* = 0.004)

After being adjusted for diameter stenosis, total plaque density, lipid percentage, and fiber percentage, mean plaque burden was an independent predictor for the prevalence of CN in calcified plaques (OR = 1.102, 95% CI: 1.025 -1.185, *P* = 0.009). As shown in Fig. 3, the AUC was 0.753 (95% CI: 0.615–0.890, *P* = 0.004). When using 84.85% as the best cutoff value, the diagnostic sensitivity and specificity of mean plaque burden for predicting the presence of a CN within calcified plaques were 66.7% and 80.2%, respectively.

## Discussion

The major findings of the present in vivo study were (1) that serum ALP level and total plaque volume were independently associated with the prevalence of CN in CAD patients with calcified plaques, (2) plaques with CNs had more severe diameter stenosis, higher mean plaque burden, and plaque density, but lower lipid percentage and fiber percentage as compared with non-nodular calcified plaques, and (3) the mean plaque burden is an independent predictor of the prevalence of CN in calcified plaques.

Vascular calcification is an important component of atherosclerosis. It could be observed over all processes of atherosclerosis, especially in the advanced stage. Percutaneous coronary intervention (PCI) for severely calcified lesions remains challenging with increased rates of complications and in-stent restenosis. CN is a specific morphology of coronary calcification and one of the most common underlying mechanisms contributing to ACS. Coronary events caused by CN have an increased risk of event recurrence and target lesion revascularization, mainly driven by the continuous growth and protrusion of the calcified mass [4]. Xu Y et.al reported that the prevalence of CN detected by IVUS was 7% per artery and 30% per patient among the ACS cohort [5]. And they were present in both culprit and non-culprit lesions [5]. CNs were located more frequently within 40 mm from the ostial left anterior descending arteries (LAD) and left circumflex arteries (LCX), whereas they were evenly and more distally distributed within the right coronary arteries (RCA) [5]. Patients with CNs were significantly older and had more plaque volume, and more thick-cap fibroatheroma [5]. However, a more recent IVUS study reported a much lower prevalence of CN at 5.3% in ACS patients, and CN patients were more likely to have hypertension, chronic kidney disease, maintenance hemodialysis, and a history of PCI [4]. A study by coronary optical coherence tomography illustrated that CNs were detected in 4.2% of all lesions among CAD patients (including 48% ACS) [6]. In lesions with severe calcification (maximum calcium arc > 180°), 30% of ACS culprit lesions contained a CN, and the presence of a CN was associated with ACS presentation independent of other vulnerable plaque morphologies [6]. The presence of a CN was associated with severe calcification and larger hinge movement of the coronary artery (especially ostial and mid RCA) [6]. Our study population focused on CAD patients with calcified plaques. We did not find the different prevalence of cardiovascular risk factors between CN patients and those with non-nodular atherosclerotic calcification including age, hypertension, diabetes, smoking, and chronic kidney disease. Furthermore, there is no difference between the two groups in terms of clinical ACS presentation and lesion location. In the present study, CNs were identified in 11.4% of patients with calcified coronary atherosclerosis plaques and 9.4% of calcified lesions. The heterogeneity of the study population may be the substrate for the variance. The present study also showed that the total plaque volume is independently associated with the prevalence of a CN in CAD patients with calcified plaques, and the mean plaque burden is an independent predictor of the prevalence of a CN in calcified plaques at lesion level, which is similar with the findings from Xu Y [5].

It has been reported that pathology and IVUS imaging are both strikingly different between a CN and a non-nodular atherosclerotic calcification. Pathologically, CNs tended to be associated with heavily calcified plaques and there was an extremely thin fibrous cap over the nodule [7, 8]. Immunohistochemistry revealed complete endothelial cell coverage over the nodule, the partly scarce media, and fibrin meshwork within the nodule [9]. Neovascularization was observed within the loose extracellular matrix beside calcification within the nodule [9]. The distinct image of CNs on grayscale IVUS are convex luminal surface and irregular luminal surface [2]. A previous study illustrated that coronary CTA might emerge as a tool to aid in the quantification of calcium burden for procedural planning [3]. The present study examined the CTA characteristics of plaques with CN. On CTA imaging, the lesion with CN had a more severe mean diameter stenosis, higher mean plaque burden, and total plaque density, but lower lipid percentage and fiber percentage as compared with non-nodular calcification. The pathological and clinical significance of this specific CTA presentation needs further exploration.

Another important finding of the present study is a hint of association between ALP and CN. The formation mechanism of a CN remains unclear. In the present study, clinically widely used serum makers of vascular calcification including ALP, gamma-glutamyltransferase, and calcium were analyzed. Among them, ALP is independently associated with the prevalence of CN in patients with calcified coronary plaques. ALP is a membrane-bound metalloenzyme that catalyzes the hydrolysis of organic pyrophosphate, an inhibitor of vascular calcification [10]. Inorganic pyrophosphate (PPi) can powerfully inhibit passive calcium phosphate deposition [11, 12]. PPi is hydrolyzed into inorganic phosphate by serum ALP in vivo. Therefore, an increased ALP activity can promote ectopic calcification due to an imbalance between inorganic phosphate and pyrophosphate [11, 12]. It has been proven that ALP is implicated in the calcification of vascular smooth muscle cells [13]. IVUS study further revealed that elevated serum ALP level was independently associated with the presence of coronary calcification, minimum lumen area ≤ 4.0 mm^2^ and plaque burden > 70% [14]. The prognostic value of ALP has also been suggested in ACS patients and patients who underwent PCI [10, 13]. The higher ALP level was an independent predictor of mortality, myocardial infarction, and stent thrombosis. In the present study, serum ALP level was independently associated with the prevalence of CN in CAD patients with calcified plaques, giving a hint of ALP in the formation of CN.

This study had some limitations. This was a single-center, retrospective observational study with a limited patient number. A prospective randomized trial with larger sample size is needed to further confirm our findings.

## Conclusions

Plaques with CN had specific CTA imaging features from non-nodular coronary calcification. The presence of a CN was associated with a higher serum ALP level and plaque burden.

## Data Availability

The datasets used and/or analysed during the current study are available from the corresponding author on reasonable request and with permission of the First Affiliated Hospital of Xi’an Jiaotong University.

## Abbreviations

ACS: Acute coronary syndrome
ALP: Alkaline phosphatase
AUC: Area under the curve
CAD: Coronary artery disease
CI: Confidence interval
CN: Calcified nodule
CTA: Computed tomography angiography
GFR: Glomerular filtration rate
HDL-C: High-density lipoprotein cholesterol
hs-CRP: High-sensitivity C-reactive protein
HU: Hounsfield Units
IQR: Interquartile range
IVUS: Intravascular ultrasound
LAD: Left anterior descending artery
LCX: Left circumflex artery
LDL-C: Low-density lipoprotein cholesterol
LVEF: Left ventricular ejection fraction
OR: Odds ratio
PCI: Percutaneous coronary intervention
PPi: Inorganic pyrophosphate
pro-BNP: Pro-brain natriuretic peptide
RCA: Right coronary artery
ROC: Receiver operating characteristic
TIMI: Thrombolysis in myocardial infarction
